# Adipose-derived mesenchymal stem cell-derived exosomal miR-22-3p alleviates depression-like behaviors in a rat model via the FURIN/HMGB1 axis

**DOI:** 10.3389/fphar.2026.1924891

**Published:** 2026-10-13

**Authors:** Yuxin Xu, Yanqiu You, Dekai Wei, Rong Liang, Weiping Liang, Bingzheng Feng, Jibing Chen, Jia Liang, Fujun Li

**Affiliations:** 1 Guangxi University of Chinese Medicine, Nanning, China; 2 The Second Affiliated Hospital of Guangxi University of Chinese, Nanning, China; 3 Guangxi University, Nanning, China; 4 Department of Psychiatry and Psychology, People’s Hospital of Guangxi Zhuang Autonomous Region, Nanning, China; 5 Department of Anesthesiology, The Second Affiliated Hospital of Guangdong Medical University, Zhanjiang, China

**Keywords:** exosomes, FURIN/HMGB1 axis, hippocampus, intranasal administration, major depressive disorder, miR-22-3p, neuroinflammation

## Abstract

**Background:**

Major depressive disorder (MDD) is characterized by neuroinflammation and neuronal apoptosis, yet effective brain-targeted delivery strategies remain limited. Exosomes represent promising carriers capable of crossing the blood–brain barrier. This study investigated whether engineered exosomes delivering miR-22-3p by modulating the FURIN/HMGB1 axis.

**Methods:**

Serum microRNA expression was assessed in patients with MDD. A rat model of depression was established using chronic unpredictable mild stress (CUMS). miR-22-3p-engineered adipose-derived mesenchymal stem cell exosomes were administered intranasally. Behavioral tests (SPT, OFT, FST), RT-qPCR, ELISA, HE staining, and TUNEL assays were performed to evaluate neurobehavioral and molecular changes.

**Results:**

miR-22-3p was significantly downregulated in both MDD patients and CUMS rats (P < 0.01). Intranasal administration of engineered exosomes increased brain miR-22-3p levels and significantly improved depressive-like behaviors (P < 0.05 or P < 0.01). Mechanistically, treatment suppressed FURIN and HMGB1 expression, reduced proinflammatory cytokines (IL-1β, IL-6, TNF-α), and attenuated hippocampal neuronal apoptosis (P < 0.05 or P < 0.01). These effects were more pronounced than those of native exosomes.

**Conclusions:**

miR-22-3p-engineered exosomes exert antidepressant effects by inhibiting the FURIN/HMGB1-mediated neuroinflammatory and apoptotic pathways. This study highlights a novel intranasal exosome-based microRNA delivery strategy for MDD.

## 1 Introduction

Major depressive disorder (MDD) is a complex and markedly heterogeneous psychiatric disorder with a multifactorial etiology involving both genetic and environmental factors (Jo et al., 2015), and it is estimated to affect 350 million people worldwide. It is associated with increased morbidity and mortality, adverse health behaviors, loss of work productivity, and increased healthcare utilization (Benton et al., 2007). Suicide causes nearly 1 million deaths each year, equivalent to approximately 3,000 deaths per day. About 25% of individuals diagnosed with MDD are younger than 19 years, and approximately 40% of patients show an inadequate response to current treatments (Dwivedi, 2014). For this reason, the potential mechanisms underlying MDD and suicidal behavior have not yet been fully elucidated. With the accelerated pace of modern society and increasing life stress, the incidence of MDD continues to rise. According to the latest data released by the World Health Organization (WHO), MDD has become one of the leading contributors to the global burden of disease. Stress (Weavers et al., 2023), impaired neurogenesis, and abnormal synaptic plasticity constitute three interrelated core pathological factors in depression (Dwivedi, 2011; Mouillet-Richard et al., 2012). Its typical neurobiological alterations include depletion of monoamine neurotransmitters, downregulation of neurotrophic factor signaling, glucocorticoid receptor resistance, and abnormally increased levels of glutamate, corticotropin-releasing hormone, and cortisol (Carvalho et al., 2015; Jo et al., 2015). Stress is not only an important trigger for the onset of depression, but preclinical studies have also suggested that microglia play a key role in stress and depressive states. Stress reduces the expression of brain-derived neurotrophic factor (BDNF) in limbic brain structures involved in emotional regulation, whereas antidepressant treatment can reverse or block the effects of stress. Decreased levels of BDNF, together with reductions in other neurotrophic factors, may lead to atrophy in certain limbic structures, including the hippocampus and prefrontal cortex (Duman and Monteggia, 2006).

Recent evidence indicates that extracellular vesicles (EVs) secreted by neurons and glial cells, including endosome-derived exosomes and plasma membrane-derived fragments, play critical roles in intercellular communication and neuroinflammation by transporting messenger RNAs (mRNAs), microRNAs (miRNAs), and proteins (Pegtel et al., 2014). EVs are considered to participate in the pathogenesis and propagation of inflammatory diseases and also show promise as biomarkers and therapeutic carriers (Pegtel et al., 2014). Reactive microglia have been shown to release exosomes and microvesicles carrying the proinflammatory cytokine interleukin-1β (IL-1β), the IL-1β-processing enzyme caspase-1, and the P2X7 receptor, all of which may induce and propagate inflammatory responses in the brain (Frühbeis et al., 2013). In recent years, exosomes have become a major focus of research because of their unique physiological properties. The blood–brain barrier (BBB) is an essential barrier for maintaining brain homeostasis, and it prevents approximately 95% of drug molecules from entering the brain, thereby greatly limiting the development of neurotherapeutics (Dong, 2018). However, accumulating evidence suggests that exosomes can cross the BBB and exert critical regulatory effects in the pathogenesis of MDD by delivering bioactive molecules such as non-coding RNAs (ncRNAs), promoting angiogenesis, and suppressing neuroinflammatory responses. Studies have shown that miR-22-3p acts on Sox9 through the NF-κB signaling pathway, thereby improving cognitive function and reducing Aβ deposition in Alzheimer’s disease model mice (Xia et al., 2022). Overexpression of miR-22-3p inhibits cell viability and promotes apoptosis through the regulation of apoptosis-related genes, whereas silencing miR-22-3p exerts opposite effects and counteracts the effects of hf-treated exosomes. FURIN, a target gene of miR-22-3p, is negatively regulated by miR-22-3p, and FURIN overexpression can promote cell viability and inhibit apoptosis. Exosomal miR-22-3p increases the risk of myocardial injury through regulation of FURIN (Lu et al., 2023). Exosomes secreted by mesenchymal stem cells (MSCs) can carry miR-22-3p, and LGALS1 is a binding target of miR-22-3p; this interaction can inhibit the epithelial–mesenchymal transition (EMT) process in melanoma cells (Chen et al., 2022). In addition, miR-22-3p regulates the phenotypic transformation of aortic smooth muscle cells (ASMCs) by targeting CHD9, thereby inhibiting vascular remodeling and oxidative stress *in vivo* (Chen et al., 2021). miR-22-3p also suppresses inflammation and apoptosis by downregulating inflammatory proteins, including high mobility group box 1 protein (HMGB1), phosphorylated p65 (p-p65), Toll-like receptors (TLRs), and proinflammatory mediators such as interleukin-1β (IL-1β), IL-6, TNF-α, and NO (Wang et al., 2020). In the present study, a rat model of depression was established by social isolation combined with chronic unpredictable mild stress, and exosomes were delivered through intranasal administration to investigate the regulatory effects of engineered exosomes on neuroinflammation and neuroprotection. This study therefore provides experimental evidence supporting the feasibility of intranasal administration of engineered exosomes as an innovative therapeutic strategy for depression.

Neuroimmune activation plays an important role in depression by influencing disease progression through inflammatory mediators, immune cell activation, and neurotransmitter disturbances. During persistent chronic inflammation, the expression levels of proinflammatory cytokines, such as interleukin-6 and tumor necrosis factor-α, are significantly upregulated. Long-term depression can disrupt the structural integrity and functional stability of the blood–brain barrier, thereby promoting the infiltration of inflammatory mediators into the central nervous system and activating microglia. Activated microglia subsequently secrete excessive amounts of reactive oxygen species, chemokines, and proinflammatory cytokines, ultimately leading to neuroinflammation. Because the hippocampus abundantly expresses receptors for proinflammatory cytokines, sustained inflammation can damage the structure and function of neurons in this region, impair brain function, and trigger or exacerbate depression (Ali et al., 2025). Under homeostatic conditions, microglia monitor synaptic function and regulate synaptic plasticity, whereas under reactive conditions, the substances they release, such as TNF-α, brain-derived neurotrophic factor (BDNF), and IL-1β, can all affect synaptic plasticity. In addition, TNF-α is associated with homeostatic synaptic plasticity, a form of plasticity that maintains neural activity within a dynamic range through a negative feedback mechanism. Microglia can regulate TNF-α-mediated synaptic plasticity (Leonoudakis et al., 2004).

Weighted gene co-expression network analysis (WGCNA) is a bioinformatics method used to construct gene co-expression networks and identify functionally related gene modules (Tang et al., 2023; Zhu et al., 2024). WGCNA builds gene co-expression networks by calculating expression similarity among genes and applies modular analysis to identify gene sets associated with specific biological processes (BPs) or cell types (De Sadeleer et al., 2022; Zheng et al., 2023)^,^. This method has unique advantages in the study of complex diseases because it can reveal potential regulatory mechanisms and hub genes at the whole-genome leve (Tao et al., 2022; Wang et al., 2022). In recent years, WGCNA has been widely applied in studies of various diseases, including cancer, cardiovascular diseases, and neurodegenerative disorders. By systematically identifying disease-related gene modules, WGCNA provides insights into their biological functions and underlying mechanisms, thereby laying a theoretical foundation for molecular diagnosis and targeted therapy (Kanehisa et al., 2022; Lu et al., 2024; Yin et al., 2022).

d trypsin were purchased from Chinese Greatwall Shark Company. ELISA kits for IL-1β, IL-6, and TNF-α were purchased from Wuhan Elabscience Biotechnology Co., Ltd.

## 2 Methods1 materials

### 2.1 Materials

#### 2.1.1 Experimental animals

In this study, 50 specific pathogen-free (SPF) Sprague–Dawley (SD) rats were provided by Guangdong Vital River Laboratory Animal Technology Co., Ltd., with a mean body weight ranging from 210 to 240 g. The supplier license number was SCXK (Yue) 2022–0,063. The animals were housed under standardized environmental conditions, with the indoor temperature maintained at (22 ± 3) °C and the relative humidity controlled within an appropriate range of 50%–60%. A 12-h light/12-h dark cycle was implemented to ensure normal physiological rhythms. During the experiment, the animals had free access to food and water. All animal experimental procedures were conducted in accordance with the relevant guidelines of the Experimental Animal Ethics Committee of Guangxi University and were approved by the committee (Ethics Approval No. GXU-2025–375).

#### 2.1.2 Study population

This study enrolled patients with depression who were treated at the Department of Psychiatry, Guangxi Zhuang Autonomous Region People’s Hospital. The diagnosis of depression was established by experienced psychiatrists in accordance with the Diagnostic and Statistical Manual of Mental Disorders, Fifth Edition (DSM-5). The inclusion criteria were as follows: (1) meeting the diagnostic criteria for major depressive disorder; (2) aged 18–60 years; and (3) able to understand the study procedures and provide written informed consent. The exclusion criteria were as follows: (1) comorbid severe psychiatric disorders, such as schizophrenia or bipolar disorder; (2) severe neurological diseases or brain injury; (3) serious systemic diseases, including cardiac, hepatic, or renal failure; and (4) pregnancy or lactation.

All procedures involving human participants were conducted in accordance with the ethical standards of the Ethics Committee of Guangxi Zhuang Autonomous Region People’s Hospital and complied with the Declaration of Helsinki and its subsequent amendments. The study protocol was approved by the institutional ethics committee (Approval No. KY-ZC-2021–32). Written informed consent was obtained from all participants prior to enrollment. All samples were anonymized before analysis to ensure participant privacy. Peripheral blood samples were collected from all participants under standardized conditions, and serum was isolated for subsequent experimental analyses.

#### 2.1.3 Major reagents and instruments

Major instruments: Cell and molecular biology platform: CO_2_ incubator (Guangzhou Hucheng DF12), biosafety cabinet, inverted fluorescence microscope (Leica DMI8), gradient PCR system (Bio-Rad T100), real-time fluorescence quantitative PCR system (Bio-Rad CFX Connect), flow cytometer (Beckman CytoFLEX), high-speed refrigerated centrifuge (Thermo ST16R), automatic tissue dehydrator (Leica ASP300S), embedding machine (Leica, EG1160), and others. Animal behavior and imaging platform: small-animal anesthesia machine, Xinruan behavioral testing system, open field test apparatus, and small-animal *in vivo* imaging system (PerkinElmer IVIS Lumina LT). Histomorphology platform: fully automatic microtome (Thermo HM355S), paraffin microtome (Thermo FINESSEE+), automatic staining and coverslipping machine, upright biological microscope (Olympus BX63), and image acquisition system.

Major reagents: exosomes (Ruikang Hospital Affiliated to Guangxi University of Chinese Medicine); DiR fluorescent labeling kit (Mlinked Biotech, E0039); TUNEL kit (Elabscience, E-CK-A320); 4% paraformaldehyde (Longke Chemical Co., Ltd.); RT-qPCR kit (Wuhan Servicebio Technology Co., Ltd.); OCT embedding compound (SAKURA, Japan); PBS; fetal bovine serum (BI, Australia); DMEM medium an.

All experimental procedures were performed in accordance with relevant institutional guidelines. This study was reported in compliance with the ARRIVE guidelines 2.0.

### 2.2 Serum microRNA expression analysis

Peripheral blood samples were collected from all participants under standardized conditions, and serum was separated for subsequent analyses. The study protocol was approved by the Ethics Committee of Guangxi Zhuang Autonomous Region People’s Hospital (Approval No. KY-ZC-2021–32), and written informed consent was obtained from all participants. Total RNA was extracted from serum samples and reverse transcribed into complementary DNA (cDNA). RT-qPCR was performed using a SYBR Green RT-qPCR kit on a CFX96 Touch Real-Time PCR System. The expression levels of microRNAs, including miR-22-3p, miR-142, and miR-204, were quantitatively measured. U6 was used as the internal reference gene, and the relative expression levels of each microRNA were calculated using the 2^−ΔΔCt method. The primer sequences are listed in Table 1.

**TABLE 1:** RT-qPCR primer sequences and annealing temperatures.

Primer name	miRBase	Primer sequence (5‘-3‘)	Length (bp)
miRNA-22-3 pF	MI0000078	GCT​GAG​CCG​CGT​AGT​TCT​T	19
miRNA-22-3 pR		GGC​AGA​GGG​CAA​CAT​TTC​TT	20
miRNA-142-5 pF	MI0000458	GCG​CGC​ATA​AAG​TAG​AAA​GC	20
miRNA-142-5 pR		GCT​GTC​AAC​GAT​ACG​CTA​CG	20
miRNA-144-3 pF	MI0000460	GGAUCAUCAUACUAAG	16
miRNA-144-3 pR		UAC​AGU​AUA​GAU​GUG​UAC​U	19
miRNA-204-5 pF	MI0000265	GAC​GCT​TTC​CCT​TTG​TCA​TCC​T	22
miRNA-204-5 pR		GTG​CAG​GGT​CCG​AGG​TAT​TC	20
U6-S	-	CTCGCTTCGGCAGCACA	17
U6-A	-	AAC​GCT​TCA​CGA​ATT​TGC​GT	20

### 2.3 Acquisition of the target genes of engineered exosomal miRNA-22-3p-EXOs

#### 2.3.1 Identification of major depressive disorder-related targets

Using “Major Depressive Disorder” as the keyword, disease-related targets were retrieved from six databases, including GeneCards (http://www.genecards.org/), DrugBank (http://go.drugbank.com), OMIM (http://omim.org/), DisGeNET (https://disgenet.com/plans), PharmGKB (http://www.clinpgx.org), and TTD (http://ttd.idrblab.cn), followed by removal of duplicate entries. All disease-related genes were then integrated, and a Venn diagram was generated using R software.

#### 2.3.2 Construction and analysis of the miRNA-22-3p-EXOs–MDD network

Using miRNA-22-3p-EXOs and the intersecting target genes as nodes, a “miRNA-22-3p-EXOs–MDD” network model was constructed using Cytoscape 3.10.3 to identify the potential common targets through which miRNA-22-3p-EXOs may exert therapeutic effects on major depressive disorder.

#### 2.3.3 Construction of the protein–protein interaction (PPI) network and identification of core targets

The targets of miRNA-22-3p-EXOs were intersected with those associated with major depressive disorder to obtain common targets. A PPI network was then constructed using the STRING database with the following settings: species was set as “*Homo sapiens*,” the minimum required interaction score was set at >0.900, and isolated protein nodes without interactions were removed to obtain a high-quality PPI network with strong relevance. In addition, Cytoscape software was used to construct a “core component–core target–core pathway” network. The network was scored using the CytoNCA (MCODE) plug-in, and nodes with scores above the median were retained for subnetwork analysis.

#### 2.3.4 Gene Ontology (GO) functional annotation and Kyoto Encyclopedia of genes and genomes (KEGG) pathway enrichment analysis

R software was used to analyze the IDs corresponding to the intersecting genes, followed by GO and KEGG enrichment analyses, with P value used as the filtering criterion. The results were visualized on the BIOINF platform (https://www.bioinformatics.com.cn/). On the plots, the x-axis represents the ratio of the number of genes from the selected gene set enriched in a given pathway to the number of background genes enriched in that pathway; therefore, a larger ratio indicates that more genes were enriched in that pathway. The y-axis represents the names of the enriched pathways, ranked in ascending order according to P value. The size of each dot indicates the gene count, with larger dots representing a greater number of genes enriched in the pathway. The color gradient of the dots is particularly important, as it reflects the magnitude of the P value; a smaller P value indicates that the pathway is more significantly enriched. In addition, pathway enrichment maps were generated using R software to illustrate the distribution of target genes across different pathway maps.

#### 2.3.5 Grouping and drug administration

After 1 week of acclimatization, rats were randomly assigned into five groups (n = 10 per group): Control group, CUMS model group, Fluoxetine group (FLX + CUMS), Natural exosome group (EXO + CUMS), Engineered exosome group (miR-22-3p-EXO + CUMS). Randomization was performed using a random number method. Behavioral assessments and data analyses were conducted by investigators blinded to group allocation. No animals were excluded from the analysis.

Except for the normal group, all other groups were subjected to CUMS modeling. Drug administration in the treatment groups began in the fourth week. The exosome group received intranasal administration of 50 μL daily (containing 4 × 10^5^ exosome particles), and the engineered exosome group received intranasal administration of 50 μL daily (containing 4 × 10^5^ miRNA-22-3p-EXO particles), administered 5 days per week followed by 2 days of rest. The adult fluoxetine dose is 20–60 mg, and after conversion to the clinically equivalent dose for rats, the fluoxetine group received 0.0036 g/kg. The model group was given normal saline by gavage.

All animal procedures were approved by the Experimental Animal Ethics Committee of Guangxi University (Approval No. GXU-2025–375). All efforts were made to minimize animal suffering.

#### 2.3.6 Establishment of the depression model by social isolation combined with chronic unpredictable mild stress (CUMS)

Except for the normal group, all rats were housed individually in single cages. The depression model was established using multiple random stressors: A. water deprivation (24 h); B. forced swimming in ice water/hot water (4 °C/43 °C, 5 min); C. reversal of the light–dark cycle (24 h); D. food deprivation (24 h); E. foot shock stimulation (30 V, 60 s); F. wet bedding (24 h); G. exposure to odor stimulation; H. empty cage (24 h); and I. noise stimulation (4 h). During the experimental period (56 days), one to two stressors were randomly applied each day, and the same stressor was not repeated on two consecutive days to ensure that the experimental animals could not develop adaptive responses. Rats in the normal group were maintained under routine feeding conditions and were not exposed to the above stressors.

### 2.4 Isolation and culture of ADSCs and purification and identification of miRNA-22-3p exosomes

#### 2.4.1 Isolation and culture of ADSCs

With approval from the Ethics Committee of the Department of Plastic Surgery, Ruikang Hospital Affiliated to Guangxi University of Chinese Medicine (Approval No.: KY2023-057), human ADSCs were isolated from adipose tissue obtained from healthy donors. Written informed consent was obtained from all donors. The specific procedure was as follows: the adipose tissue was repeatedly washed with PBS containing antibiotics to remove blood and impurities, and then digested with 0.1% collagenase I in a 37 °C water bath for 30 min. After digestion was terminated, an equal volume of DMEM medium containing 10% fetal bovine serum was added. Following centrifugation, the supernatant was discarded and the pellet was collected. After filtration through a 70 μm cell strainer to remove tissue debris, the cells were resuspended in human mesenchymal stem cell culture medium, seeded into culture flasks, and maintained under adherent culture conditions in a 37 °C, 5% CO_2_ incubator. Cells were passaged when confluence reached 80%–90%, and passages 3 to 8 were used for subsequent experiments. Under the microscope, the cells showed spindle-shaped adherent growth, uniform morphology, and typical characteristics of ADSCs.

#### 2.4.2 Purification and identification of miRNA-22-3p exosomes

Passages 3–8 ADSCs were transfected with a lentiviral vector to overexpress miRNA-22-3p, thereby generating ADSCs-miRNA-22-3p cells; non-transfected cells were used as the negative control (NC-ADSCs). At 48 h after transfection, the culture medium was replaced, and the cells were further cultured to the logarithmic growth phase, after which the cell supernatant was collected. Exosomes were isolated by tangential flow filtration (TFF) combined with chromatographic purification using a commercial exosome extraction system (Huakan Biotechnology Co., Ltd., Beijing). The obtained exosomes were sterilized by filtration through a 0.22 μm membrane to obtain NC-Exos and miRNA-22-3p-modified exosomes, which were aliquoted and stored at −80 °C for later use.

Transmission electron microscopy was used to observe exosomes: 10 μL of exosome suspension was dropped onto a Formvar-carbon copper grid and allowed to stand at room temperature for 1 min. The excess liquid was carefully removed from the edge using filter paper. Then, 10 μL of uranyl acetate staining solution was added onto the copper grid and stained for 1 min in the dark. The staining solution was removed with filter paper, and the grid was dried at room temperature for 5 min. Images were then acquired under a transmission electron microscope at an accelerating voltage of 100 kV.

Analysis of exosome particle size and concentration: 10 μL of exosome suspension was diluted with PBS to 30 mL for later use. Before sample loading, the nanoflow cytometer was validated using standard particles to confirm acceptable performance. Then, 20 μL of the original exosome suspension was diluted with PBS to a final volume of 1 mL and analyzed using the nanoflow cytometer to determine particle size distribution.

Detection of exosome markers: exosome markers CD9, CD63, and CD81 were detected by nanoflow cytometry.

### 2.5 Behavioral experiments

#### 2.5.1 Body weight

After the adaptive feeding period, body weight was measured in each group on days 0, 7, 14, 21, 28, 35, 42, 49, and 56. Body weight was used as a key indicator reflecting feeding status, and weight loss may suggest depression-related behavioral manifestations in rats, such as anorexia, low mood, reduced motivation, and emaciation.

#### 2.5.2 Open field test (OFT)

The open field test was performed on day 56 using an uncovered black box (100 cm × 100 cm × 40 cm) as the testing apparatus. The floor of the box was divided into 16 equal square areas. The experiment was conducted in a quiet laboratory with uniform lighting and without external interference. Before the test, the rats were allowed to acclimate to the experimental environment for 60 min to reduce stress caused by environmental changes. Each rat was placed individually in the center of the arena and allowed to move freely for 5 min, while its locomotor trajectory was recorded using the VisuTrack video tracking system. By analyzing the time spent in the central area and the number of entries into the center, the spontaneous locomotor activity and anxiety-like behavioral changes of the rats were evaluated.

#### 2.5.3 Sucrose preference test (SPT)

Step 1: Rats were housed individually in cages for sucrose drinking training. During the first 24 h, two bottles containing 1%–2% sucrose solution were provided. During the following 24 h, one bottle contained 1%–2% sucrose solution and the other contained ordinary drinking water, and the positions of the two bottles were exchanged during this period.

Step 2: Measurement of the sucrose preference index: after 24 h of food and water deprivation, the sucrose preference index was measured. During the test, the volume consumed from the two bottles within 2 h was recorded. The sucrose preference index was calculated as follows: sucrose preference (%) = sucrose intake/(sucrose intake + water intake) × 100%. A decrease in sucrose preference usually indicates the occurrence of anhedonia in rats, that is, a reduced ability to experience pleasure.

#### 2.5.4 Forced swimming test (FST)

Step 1: Before the experiment, the water temperature was maintained at 23 °C–25 °C. The water depth was adjusted so that the rat’s tail remained at a certain distance from the bottom of the test cylinder.

Step 2: Forced swimming test: pre-swimming was conducted 1 day before the formal experiment. On the second day, the formal forced swimming test was performed for 5 min, and the immobility time, swimming time, and climbing time of the rats were recorded. When the experimental animal stopped struggling and remained motionless or made only slight movements, it was considered immobile. After each rat completed the swimming test, the water was replaced before the next rat was tested. After the experiment, the rats were assisted in recovering body temperature.

All behavioral experiments were conducted in a controlled environment. Observers were blinded to treatment groups.

### 2.6 *In vivo* distribution of exosomes

Exosomes were labeled with near-infrared fluorescence using a DiR exosome fluorescent labeling kit (Mlinked Biotech, E0039). Briefly, 100 μg of exosome sample was prepared according to the manufacturer’s instructions. A 100 μM DiR working solution was prepared by diluting the DiR stock solution with the supplied dilution buffer. Exosomes and the working solution were mixed at a volume ratio of 10:1 and incubated for 30 min at room temperature in the dark. Unbound DiR dye was removed using the exosome free-dye removal centrifuge column provided in the kit (Mlinked Biotech, E0015). The purified DiR-labeled exosomes were resuspended in PBS and adjusted to a final volume of 200 μL.

Under light isoflurane anesthesia, DiR-labeled EXO or DiR-labeled miR-22-3p-EXO was administered intranasally to rats. Unlabeled exosomes were used as the control. Fluorescence signals were detected at 0, 12, and 24 h after intranasal administration using a small-animal *in vivo* imaging system with excitation/emission wavelengths of 748/780 nm. All imaging parameters were kept consistent. Brain fluorescence signals were measured and background-corrected, and the results were expressed as relative fluorescence intensity.

### 2.7 RT-qPCR analysis of FURIN and HMGB1 mRNA expression in rat hippocampus following treatment with miR-22-3p-modified exosomes

After behavioral testing, rats were anesthetized with isoflurane (induction 3%–5%, maintenance 1%–2%). Animals were euthanized by overdose of isoflurane followed by cervical dislocation to ensure death. The brains were rapidly removed, and hippocampal tissues were carefully isolated. Total RNA was extracted from the hippocampus, and complementary DNA (cDNA) was synthesized using a reverse transcription kit. Quantitative real-time PCR (RT-qPCR) was performed using a SYBR Green PCR kit on a CFX96 Touch Real-Time PCR Detection System (Bio-Rad, United States). GAPDH was used as the internal reference gene to normalize the mRNA expression levels of FURIN and HMGB1. Relative gene expression levels were calculated using the 2^−ΔΔCt method. Primer specificity was validated by melting curve analysis. The primer sequences and annealing temperatures are shown in Table 2.

**TABLE 2:** RT-qPCR primer sequences and annealing temperatures.

Primer name	Primer sequence (5‘-3‘)	Length (bp)
HMGB1-F	CGG​AGA​AAC​TTC​AGA​CCG​GA	20
HMGB1-R	CCC​ATG​TTT​AGT​TGA​TTT​TCC​AGC	24
FURIN-F	TGA​CAA​CAG​GCA​CGG​CAC​TC	20
FURIN-R	TAC​ACC​TAC​ACC​ACA​GAC​ACC​ATT​G	25
GAPDH-S	AGT​GCC​AGC​CTC​GTC​TCA​TA	20
GAPDH-A	GAT​GGT​GAT​GGG​TTT​CCC​GT	20

### 2.8 ELISA detection of inflammatory cytokine levels in rat hippocampal tissue

After the behavioral assessment, three rats were randomly selected from each group. The rats were anesthetized and sacrificed, and the hippocampal tissue was rapidly isolated on ice. After rinsing with 0.9% sodium chloride solution, the tissue was homogenized and centrifuged at 4 °C and 3,000 × g for 10 min, and the supernatant was collected. The supernatants and standards were processed according to the instructions of the ELISA kits. A volume of 100 μL was added to each well and incubated at 37 °C for 30 min, followed by washing with washing buffer. Then, 50 μL of chromogenic reagent A and 50 μL of chromogenic reagent B were added sequentially to each well. After incubation at room temperature in the dark, 50 μL of stop solution was added and mixed immediately. The absorbance value was read at 450 nm using a microplate reader, and the levels of IL-1β, IL-6, and TNF-α were calculated. Each experiment was independently repeated at least three times.

### 2.9 Histopathology and apoptosis assessment

#### 2.9.1 HE staining

After completion of all behavioral experiments, the rats were deeply anesthetized with isoflurane. Following cardiac perfusion with PBS and 4% paraformaldehyde, the whole brain was removed and further fixed in the same fixative for 24 h. Brain tissues containing the hippocampus and prefrontal cortex were then dissected, dehydrated through a graded ethanol series, embedded in paraffin, and sectioned at a thickness of 5 μm for HE staining. Finally, neuronal morphology and tissue structure in the hippocampus and prefrontal cortex were observed under a light microscope to evaluate pathological damage.

#### 2.9.2 TUNEL staining

Paraffin sections of brain tissue obtained 24 h after perfusion fixation were deparaffinized, rehydrated, and digested with proteinase K, and then processed according to the instructions of the TUNEL kit. TUNEL reaction mixture was added, followed by incubation at 37 °C in the dark for 60 min. After washing with PBS, the sections were mounted with a mounting medium containing DAPI. The sections were observed under a fluorescence microscope. TUNEL-positive nuclei showed green fluorescence, whereas DAPI labeled all nuclei. Fields were selected in the dentate gyrus of the hippocampus, and the apoptosis rate was calculated.

### 2.10 Statistical analysis

All quantitative data are expressed as mean ± standard deviation (Mean ± SD), and statistical analyses were performed using GraphPad Prism 10.1.2 software. Data were first tested for normality. For data conforming to a normal distribution, Student’s t-test was used for comparisons between two groups, and one-way or two-way analysis of variance (ANOVA) was used for comparisons among multiple groups. For data not conforming to a normal distribution, nonparametric tests were applied. A value of P < 0.05 was considered statistically significant, indicating that differences were regarded as statistically meaningful when P < 0.05. No protocol registration was performed for this study.

## 3 Results

### 3.1 Serum microRNA expression analysis

RT-qPCR analysis demonstrated that the expression levels of several microRNAs were significantly altered in the serum of patients with major depressive disorder (MDD) compared with healthy controls. Specifically, miR-22-3p was significantly downregulated in MDD patients *(P < 0.001*), whereas miR-142 was significantly upregulated (*P < 0.01*). In addition, miR-204 was markedly increased in the serum of MDD patients (*P < 0.001*) (Figure 1). These results indicate that these microRNAs are aberrantly expressed in the serum of patients with MDD, suggesting their potential involvement in the pathogenesis of the disorder and their value as potential biomarkers.

**FIGURE 1:**
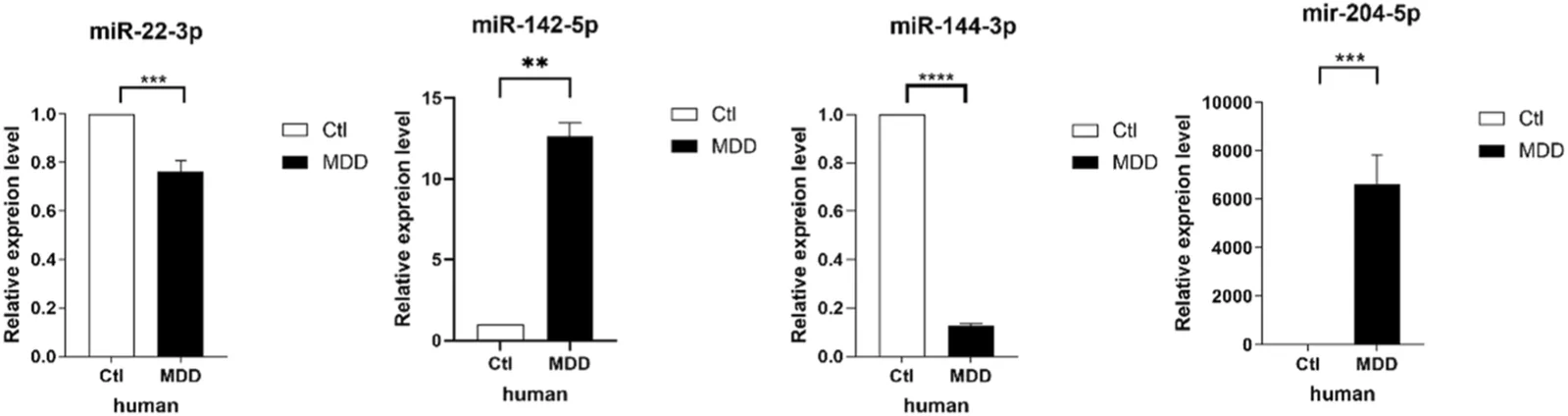
Expression levels of miR-22-3p, miR-142, and miR-204 in the serum of patients with major depressive disorder (MDD). Data are presented as mean ± SD. **P < 0.01, ***P < 0.001.

### 3.2 Screening of common targets between major depressive disorder and miRNA-22-3p-EXOs

Using “Major Depressive Disorder” as the keyword, searches were conducted in six databases, including GeneCards, DrugBank, OMIM, DisGeNET, PharmGKB, and TTD (as shown in Figures 2A,B). After database threshold screening, all retrieved disease target names were compiled and deduplicated, resulting in a total of 1,078 disease-related targets. The 1,078 disease targets and 1,948 drug-related targets were imported into Venny 2.1.0, and 162 intersecting targets were identified (as shown in Figure 2F). These common targets were considered potential therapeutic targets of miRNA-22-3p-EXOs in the treatment of major depressive disorder.

**FIGURE 2:**
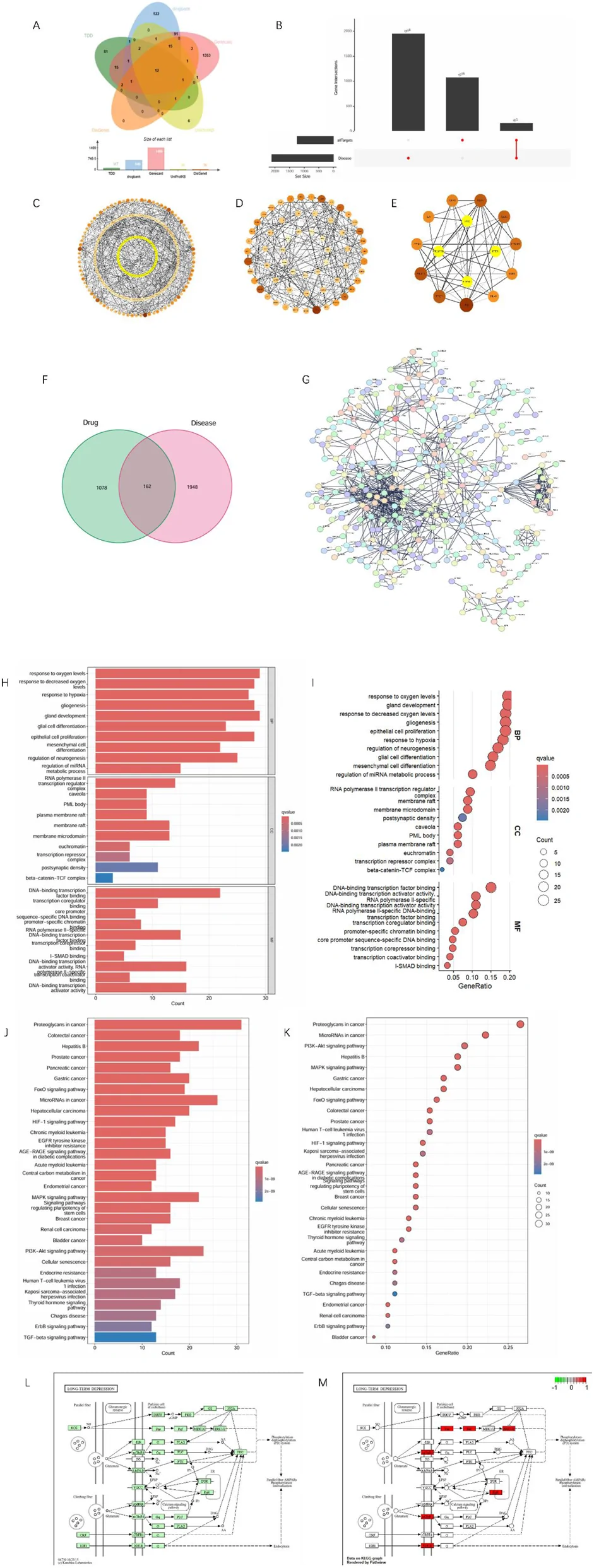
Bioinformatic analysis of miR-22-3p-EXO–MDD-related targets. **(A)** Integrated Venn diagram of MDD-related genes retrieved from multiple databases. **(B)** Quantitative comparison of disease-related genes from different databases. **(C–E)** Core PPI network maps of miR-22-3p-EXO–MDD targets. **(F)** Venn diagram showing the intersection between MDD-related genes and miR-22-3p-related targets. **(G)** Disease-related protein–protein interaction network. **(H,I)** Bar plot and bubble plot of GO functional enrichment analysis for the intersecting targets. **(J,K)** Bar plot and bubble plot of KEGG enrichment analysis. **(L)** Original KEGG pathway map of long-term depression (hsa04730) retrieved from the KEGG database. **(M)** Differential expression overlay of miR-22-3p-EXO-MDD-related targets mapped onto the long-term depression pathway (hsa04730). Red indicates upregulated genes and green indicates downregulated genes. This figure was modified from the KEGG pathway database (hsa04730), Kanehisa Laboratories.

### 3.3 Construction of the PPI network and identification of core targets

The 162 intersecting targets obtained above were imported into STRING to construct a PPI network. The specific settings were as follows: species was set as “*Homo sapiens*,” the minimum required interaction score was set at >0.900, and isolated protein nodes without interactions were removed to obtain a high-quality PPI network with strong relevance (as shown in Figures 2C–E). In addition, Cytoscape software was used to construct a “core component–core target–core pathway” network. Using the CytoNCA (MCODE) plug-in, degree, betweenness, and closeness values were calculated separately. Genes with degree, betweenness, and closeness values all greater than their respective median values were screened, and the subnetwork was further analyzed.

### 3.4 GO functional enrichment analysis and KEGG pathway enrichment

R software was used to analyze the IDs corresponding to the intersecting genes, followed by GO and KEGG enrichment analyses, with P value used as the filtering criterion. The x-axis represents the ratio of the number of genes from the selected gene set enriched in a given pathway to the number of background genes enriched in that pathway; therefore, a larger ratio indicates a greater number of genes enriched in that pathway. The y-axis shows the names of the enriched pathways, ranked in ascending order according to P value. The size of each dot indicates the gene count, with larger dots representing a greater number of genes enriched in the pathway. The color gradient of the dots reflects the magnitude of the P value, with smaller P values indicating more significant pathways (as shown in the Figures 2H–K). In addition, enrichment pathway maps were generated using R software to illustrate the distribution of target genes across different pathway maps (as shown in the Figures 2L,M).

For GO functional enrichment analysis, the top 10 results ranked by P value were visualized. In the biological process category, the main enriched terms included response to oxygen levels, response to decreased oxygen levels, response to hypoxia, gliogenesis, gland development, glial cell differentiation, epithelial cell proliferation, mesenchymal cell differentiation, regulation of neurogenesis, and regulation of miRNA metabolic process. In the cellular component category, the main enriched terms included RNA polymerase II transcription regulator complex, membrane raft, and membrane microdomain. In the molecular function category, DNA-binding transcription factor binding and DNA-binding transcription activator activity, RNA polymerase II-specific were highly associated with the treatment of depression (as shown in the Figures 2H,I).

KEGG analysis was performed on the targets of miRNA-22-3p-EXOs involved in major depressive disorder (as shown in the Figures 2J,K). The analysis showed that the related human diseases included colorectal cancer and others; the involved environmental information processing pathways included the FoxO signaling pathway, microRNAs in cancer, the PI3K-Akt signaling pathway, and the HIF-1 signaling pathway; and the involved metabolic processes included central carbon metabolism in cancer.

### 3.5 Intranasally administered labeled exosomes successfully entered the brain in rats

DiR fluorescently labeled EXO and miR-22-3p-EXO were intranasally administered to normal rats. After administration, the distribution of exosomes at 0 h, 12 h, and 24 h was observed by fluorescence microscopy (as shown in Figures 3, 4).

**FIGURE 3:**
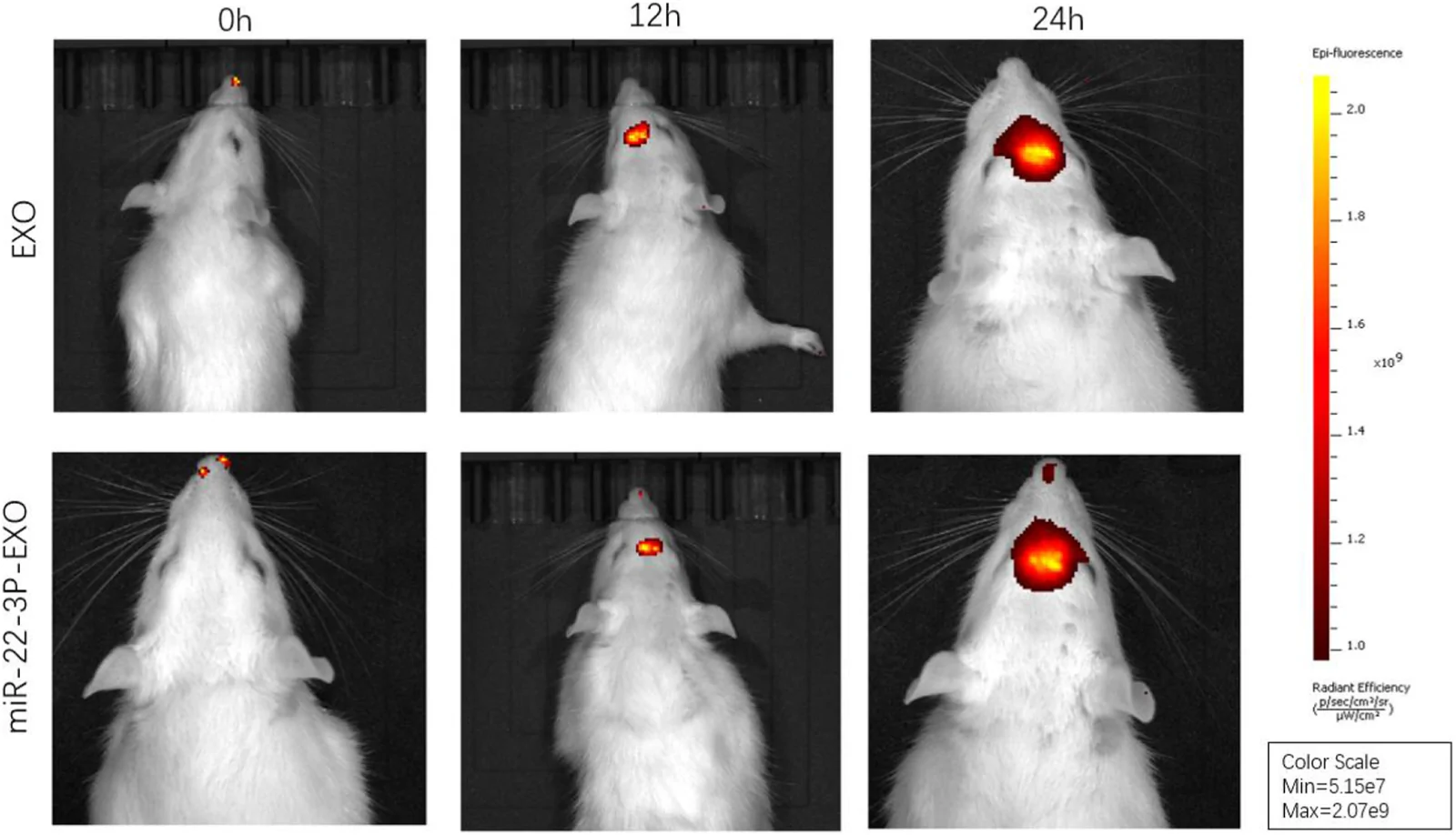
Distribution of exosomes in the rat brain.

**FIGURE 4:**
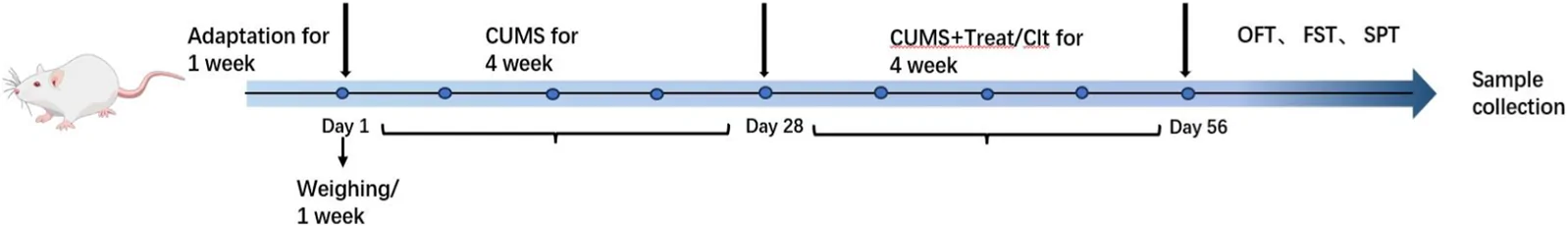
Establishment of a depressive rat model and experimental protocol.

### 3.6 Purification and identification of ADMSCs-EXO and transfected miR-22-3p-EXO

Results of exosome morphology, particle size, and protein characterization showed that exosomes were successfully isolated by tangential flow filtration combined with chromatographic purification. Transmission electron microscopy showed that the exosomes exhibited typical cup-shaped morphology with a bilayer membrane, with an average particle size of 79 nm and a predominant particle size of 60 nm. BCA assay showed that the protein concentration of the exosomes was 44.72 mg/mL (as shown in Figures 5A–D,F). For relative miRNA expression analysis, EXO-miRNA samples were used as the control group, and the relative expression of miR-22-3p in transfected exosomes was increased by 15.7-fold (as shown in Figure 5E). Microbiological testing, *mycoplasma* testing, and endotoxin detection were all negative, indicating that the extracted exosomes had high purity and met the basic characteristics of extracellular vesicles. Taken together, these results confirmed that the isolated nanoparticles had intact vesicular morphology, a uniform particle size distribution, and defined protein markers, consistent with the fundamental characteristics of exosome-like nanoparticles, and were therefore suitable for subsequent function studies.

**FIGURE 5:**
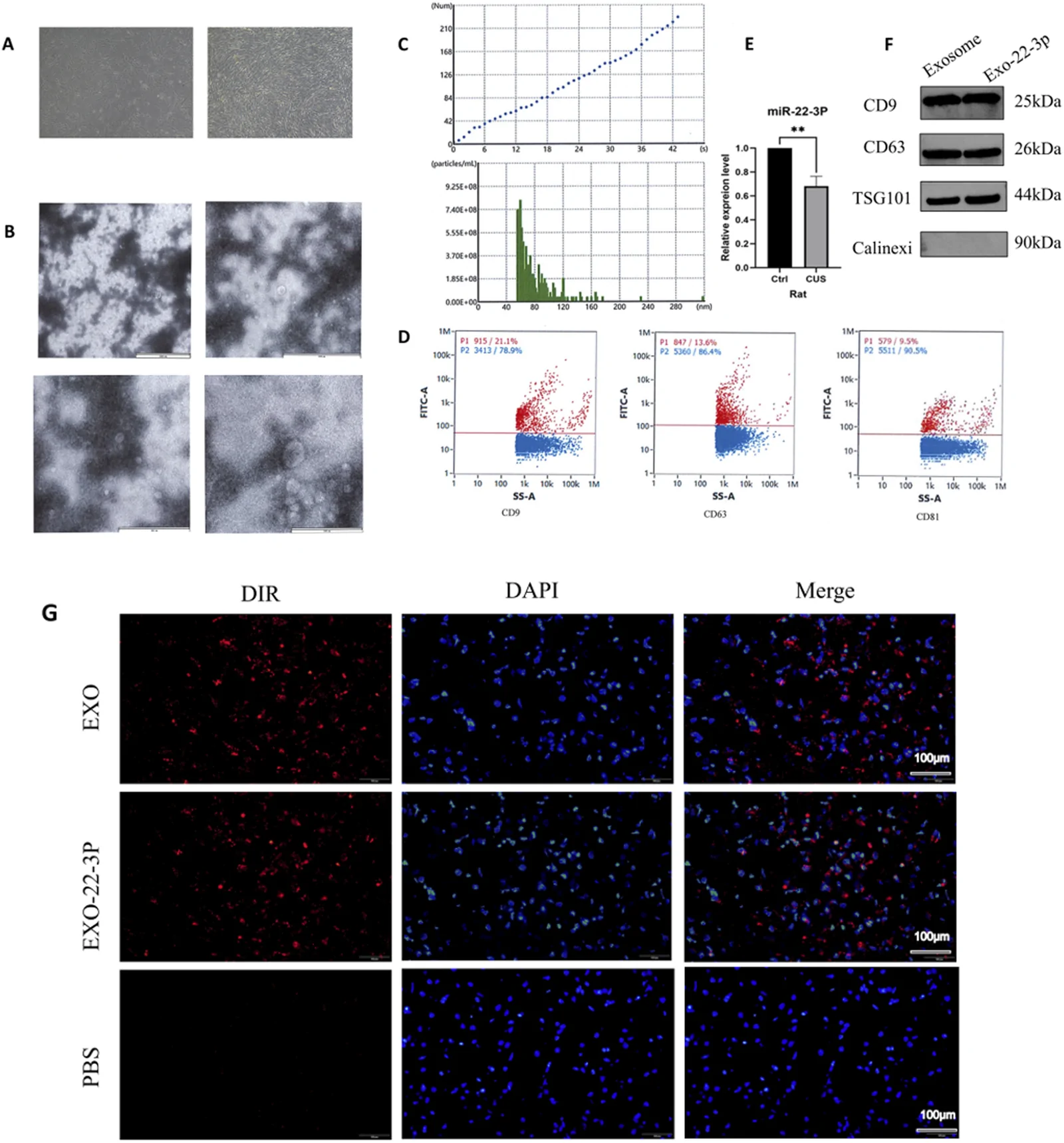
**(A)** As shown in the Figure, ADMSCs exhibited a spindle-shaped morphology and were arranged in clustered, fish school-like patterns. **(B)** Under transmission electron microscopy, the morphology of miRNA-22-3p-EXOs was observed at scales of 1,000, 600, 400, and 200 nm. **(C)** Schematic diagram of the particle size and particle concentration of miRNA-22-3p-EXOs. **(D)** Flow cytometric analysis of exosome surface markers (CD9, CD63, and CD81). **(E)** Comparison of miR-22-3p expression in hippocampal tissues between CUMS rats and normal rats. **P < 0.01. **(F)** Western blot validation of exosomal markers (CD9, CD63, and TSG101) and the negative marker calnexin in native exosomes and miR-22-3p-engineered exosomes. **(G)** Schematic illustration of rat brain frozen sections after administration of DiR-labeled exosomes.

### 3.7 Effects on body weight in rats subjected to social isolation combined with CUMS

Compared with the normal group, rats in the model group showed decreased body weight on day 7 of feeding, accompanied by mental lethargy, slowed responsiveness, dull coat appearance, and hair loss. During the subsequent period from day 7 to day 28, body weight gain in the model group was significantly reduced (*P < 0.01*). After treatment was initiated on day 28, rats in the fluoxetine group, natural exosome group, and engineered exosome group all showed significant improvement compared with the model group (*P < 0.05*). The engineered exosome group showed a statistically significant difference in body weight compared with the model group on day 35 (*P < 0.01*). The natural exosome group showed a statistically significant difference in body weight compared with the model group on day 49 (*P < 0.01*).

Statistically significant difference in body weight compared with the model group on day 56 (*P < 0.01*). By the end of the experiment, the improvement in body weight was significantly greater in all treatment groups than in the model group, and varying degrees of improvement were also observed in mental status and coat gloss (as shown in the Figure 6).

**FIGURE 6:**
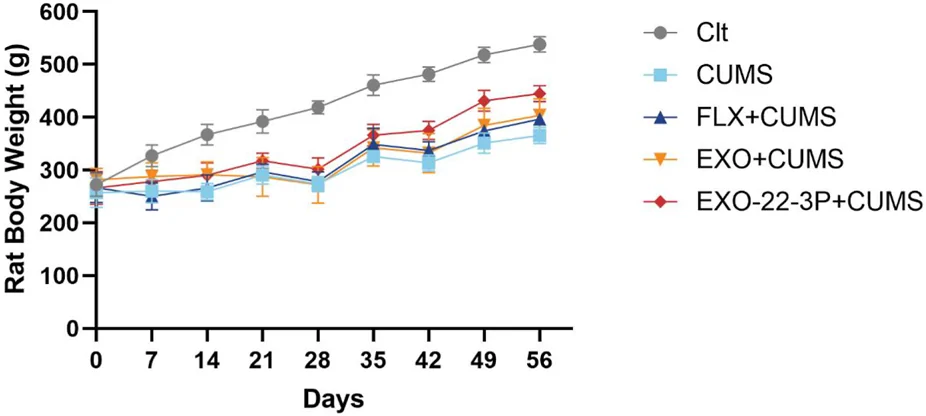
Effects of exosomes and fluoxetine on rat body weight.

### 3.8 Effects on behavioral performance in rats subjected to social isolation combined with CUMS

Behavioral tests were performed on day 56. In the sucrose preference test (SPT), compared with the normal group, the model group showed a significantly reduced sucrose preference rate (*P < 0.01*), indicating the presence of anhedonia and a diminished ability to experience pleasure. Compared with the model group, rats in the fluoxetine group, natural exosome group, and engineered exosome group all showed significant improvement (*P < 0.05*). Compared with the natural exosome group, the engineered exosome group exhibited a more pronounced improvement in sucrose preference rate (as shown in the Figures 7E,F).

**FIGURE 7:**
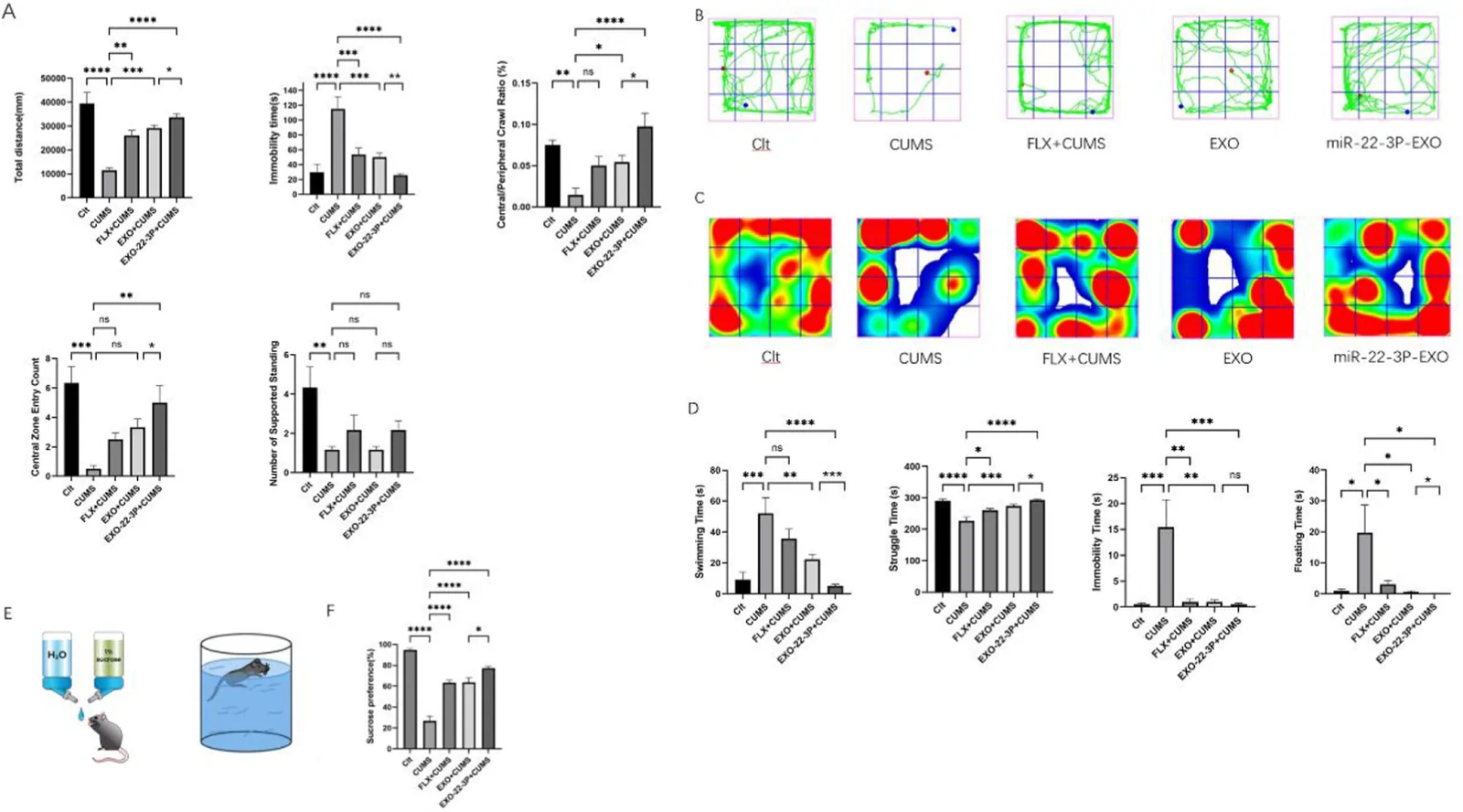
**(A)** Comparison of total distance, immobility time, central/peripheral locomotion ratio, number of entries into the central area, and number of unsupported rearings among groups in the open field test. **(B)** Representative locomotor trajectories of rats in the open field test for each group. **(C)** Heat maps of rat activity in the open field test for each group. **(D)** Comparison of swimming time, struggling time, immobility time, and floating time among groups in the forced swimming test. **(E)** Schematic diagrams of the sucrose preference test and forced swimming test in rats. **(F)** Comparison of sucrose preference among groups in the sucrose preference test. ns, P > 0.05; *P < 0.05; **P < 0.01; ***P < 0.001.

In the open field test (OFT), compared with the normal group, the model group showed reduced total locomotor distance, central/peripheral locomotion ratio, number of entries into the central area, and unsupported rearing, along with increased immobility time (*P < 0.01*). Compared with the model group, the fluoxetine group, natural exosome group, and engineered exosome group showed increased total locomotor distance and central/peripheral locomotion ratio, whereas immobility time was reduced (P < 0.01). Compared with the natural exosome group, the engineered exosome group showed statistically significant differences in total locomotor distance, central/peripheral locomotion ratio, and number of entries into the central area (*P < 0.05*) (as shown in the Figures 7A–C).

In the forced swimming test (FST), compared with the normal group, the model group showed significantly increased swimming time and immobility time (*P < 0.01*), increased floating time (*P < 0.05*), and significantly reduced struggling time (*P < 0.01*), indicating behavioral despair and abandonment of active escape attempts. Compared with the model group, rats in the fluoxetine group, natural exosome group, and engineered exosome group showed reduced swimming time and immobility time, along with increased struggling time (*P < 0.05*). Compared with the natural exosome group, the engineered exosome group showed a more marked intervention effect, with reduced swimming time (*P < 0.01*) and increased struggling time (*P < 0.05*) (as shown in the Figure 7D).

### 3.9 Effects of miR-22-3p-modified exosomes on FURIN and HMGB1 mRNA expression in the rat hippocampus

Compared with the control group, the mRNA expression levels of FURIN and HMGB1 in the hippocampus were significantly increased in the model group. Treatment with fluoxetine, native exosomes, and miR-22-3p-modified exosomes all reduced the expression levels of these genes. Notably, the reduction in FURIN expression was most pronounced in the miR-22-3p-EXO group, accompanied by a concurrent decrease in HMGB1 expression, suggesting a potential involvement of the FURIN/HMGB1-related pathway (as shown in the Figure 8).

**FIGURE 8:**
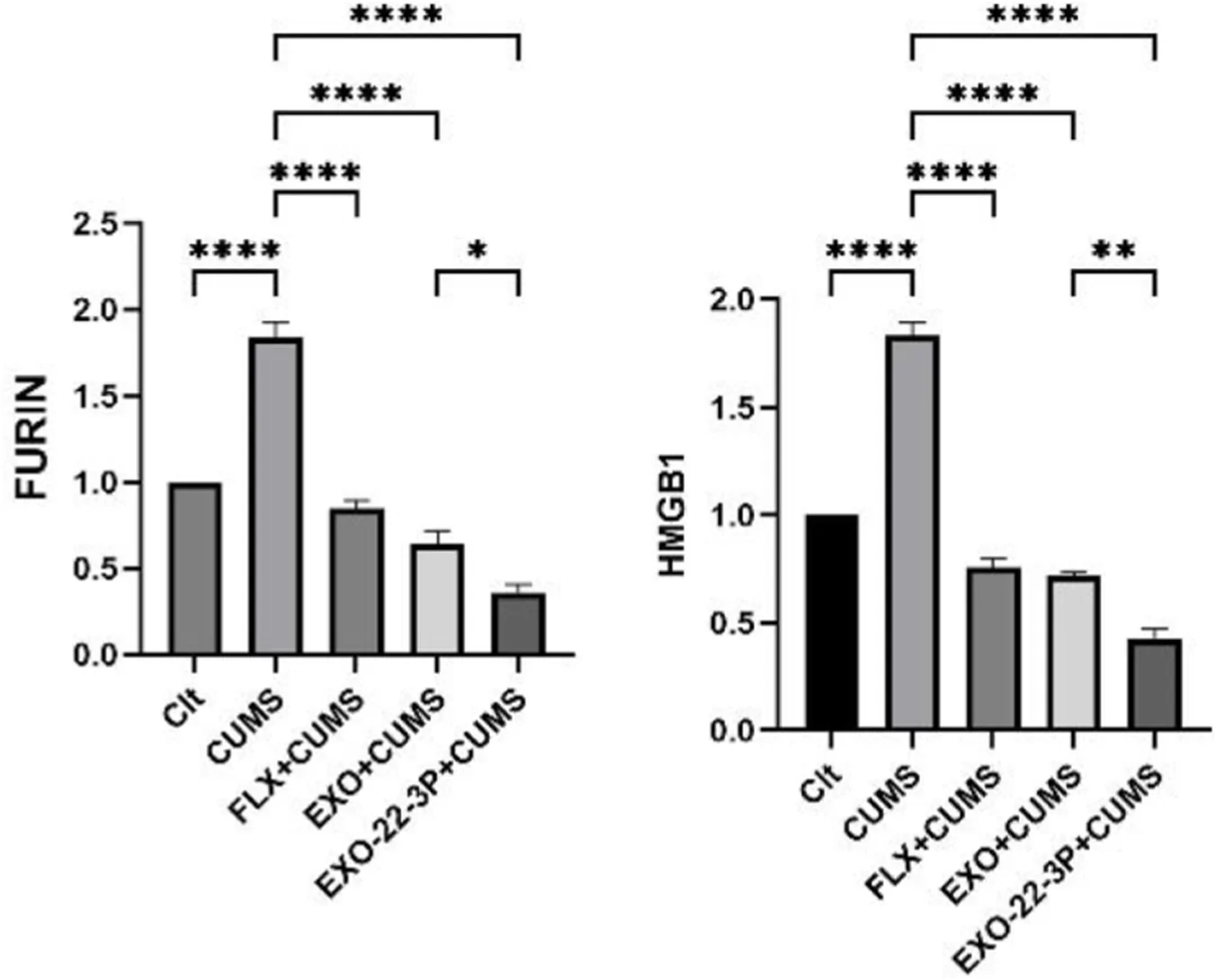
FURIN and HMGB1 mRNA expression levels. Data are presented as mean ± SD (n = 3). Statistical significance was defined as *P < 0.05, **P < 0.01, and ***P < 0.001 compared with the control group.

### 3.10 ELISA detection of IL-1β, IL-6, and TNF-α levels in the rat hippocampus

Compared with the control group (Ctr), the levels of the proinflammatory cytokines IL-1β, IL-6, and TNF-α in the hippocampal tissue of rats in the CUMS group were significantly increased, with statistically significant differences (all *P < 0.0001*), indicating that chronic unpredictable stress markedly induced inflammatory responses in the hippocampus. After intervention, the levels of inflammatory cytokines in all treatment groups generally showed a downward trend (as shown in the Figure 9).

**FIGURE 9:**
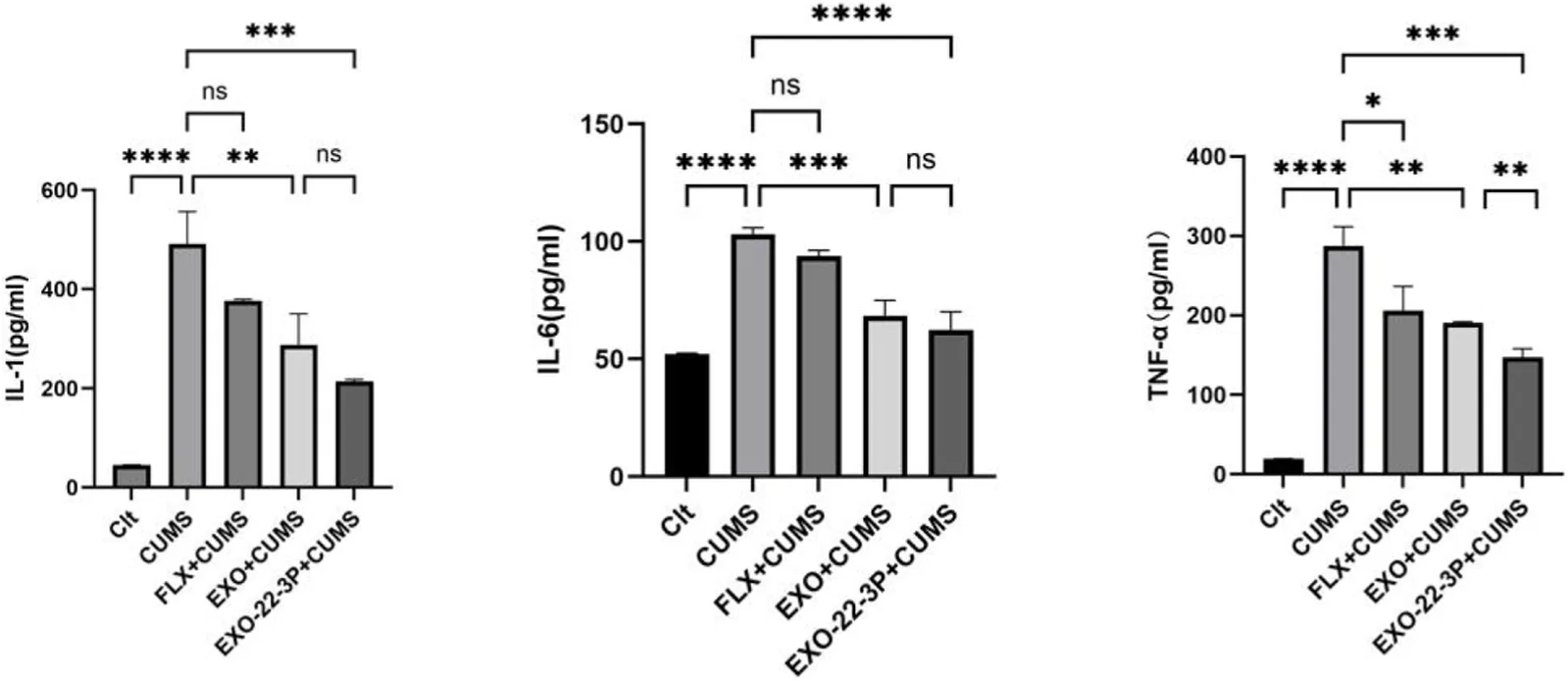
Effects of exosomes and fluoxetine on the levels of IL-1β, IL-6, and TNF-α in the rat hippocampus.

For IL-1β, compared with the CUMS group, the level in the EXO-22-3P + CUMS group was significantly decreased (*P < 0.001*). In addition, the EXO + CUMS group showed a further decrease compared with the FLX + CUMS group (*P < 0.01*), whereas there was no statistically significant difference between the EXO + CUMS group and the EXO-22-3P + CUMS group (ns).

For IL-6, compared with the CUMS group, the EXO-22-3P + CUMS group showed a significant decrease (*P < 0.0001*), whereas there was no significant difference between the FLX + CUMS group and the CUMS group (ns). Meanwhile, the EXO + CUMS group was significantly lower than the FLX + CUMS group (*P < 0.001*), and no statistically significant difference was observed between the EXO + CUMS group and the EXO-22-3P + CUMS group (ns).

For TNF-α, compared with the CUMS group, the FLX + CUMS group already showed a significant decrease (P < 0.05), and the decrease in the EXO-22-3P + CUMS group was more pronounced (*P < 0.001*). In addition, the EXO + CUMS group was further reduced compared with the FLX + CUMS group (*P < 0.01*), and the EXO-22-3P + CUMS group was also further reduced compared with the EXO + CUMS group (*P < 0.01*).

Taken together, both exosome treatment and fluoxetine intervention were able to downregulate the expression of CUMS-induced inflammatory cytokines in the rat hippocampus to a certain extent, among which EXO-22-3P treatment showed a more pronounced effect in ameliorating hippocampal inflammation.

### 3.11 Hippocampal neuronal apoptosis in the rat model induced by social isolation combined with CUMS and the neurorestorative effects of engineered exosomes

1. HE staining was used to examine morphological changes in neurons in the hippocampus and prefrontal cortex of rats in the five groups. The results showed that, in the normal group, hippocampal neurons were densely and compactly arranged, with full cytoplasm, tight intercellular spaces, and no cellular edema. Compared with the normal group, the model group showed a marked reduction in neuronal number, disordered cell arrangement, deeply stained nuclei, and interstitial edema and loosening. Neurons exhibited karyolysis, pyknosis, and fragmentation, and some cells collapsed with loss of structural integrity. In the EXO-22-3P exosome group, neuronal morphology was relatively preserved, the number of neurons increased, and staining became clearer (as shown in the Figure 10).
2. TUNEL Staining

**FIGURE 10:**
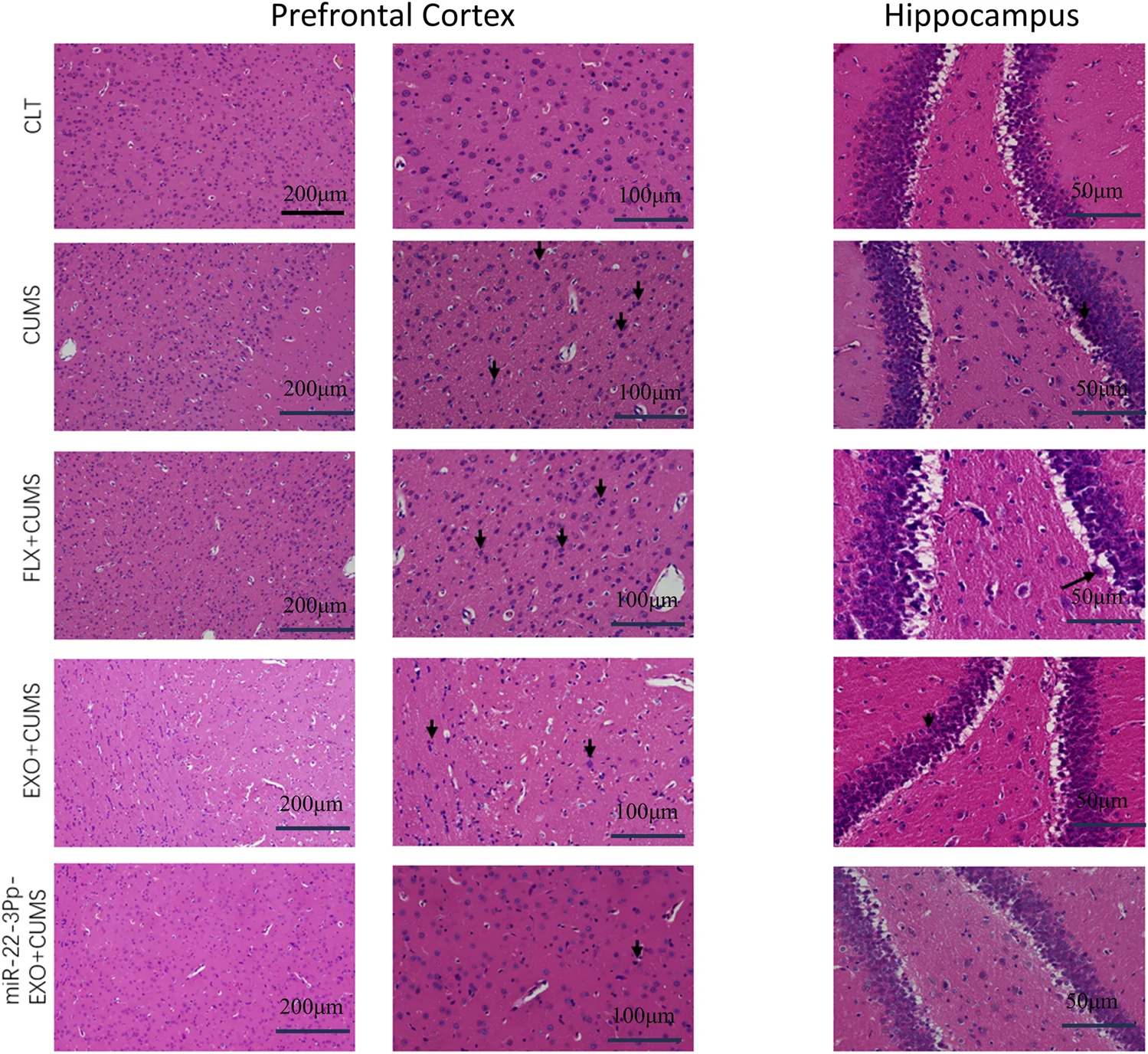
Effects of Exosomes and Fluoxetine on Pathological Alterations in the Rat Hippocampus and Prefrontal Cortex. HE staining of hippocampal tissue (×400) and prefrontal cortex (×100 and ×200) in rats from each group.

TUNEL staining was further performed to evaluate neuronal apoptosis in the hippocampal tissues of rats from the five groups.

Compared with the control group, the model group showed a significantly increased neuronal apoptosis rate (P < 0.01). Compared with the model group, the exosome group showed a reduced apoptosis rate (P < 0.05), while the engineered exosome group exhibited a more significant decrease in neuronal apoptosis (P < 0.01) (as shown in the Figure 11).

**FIGURE 11:**
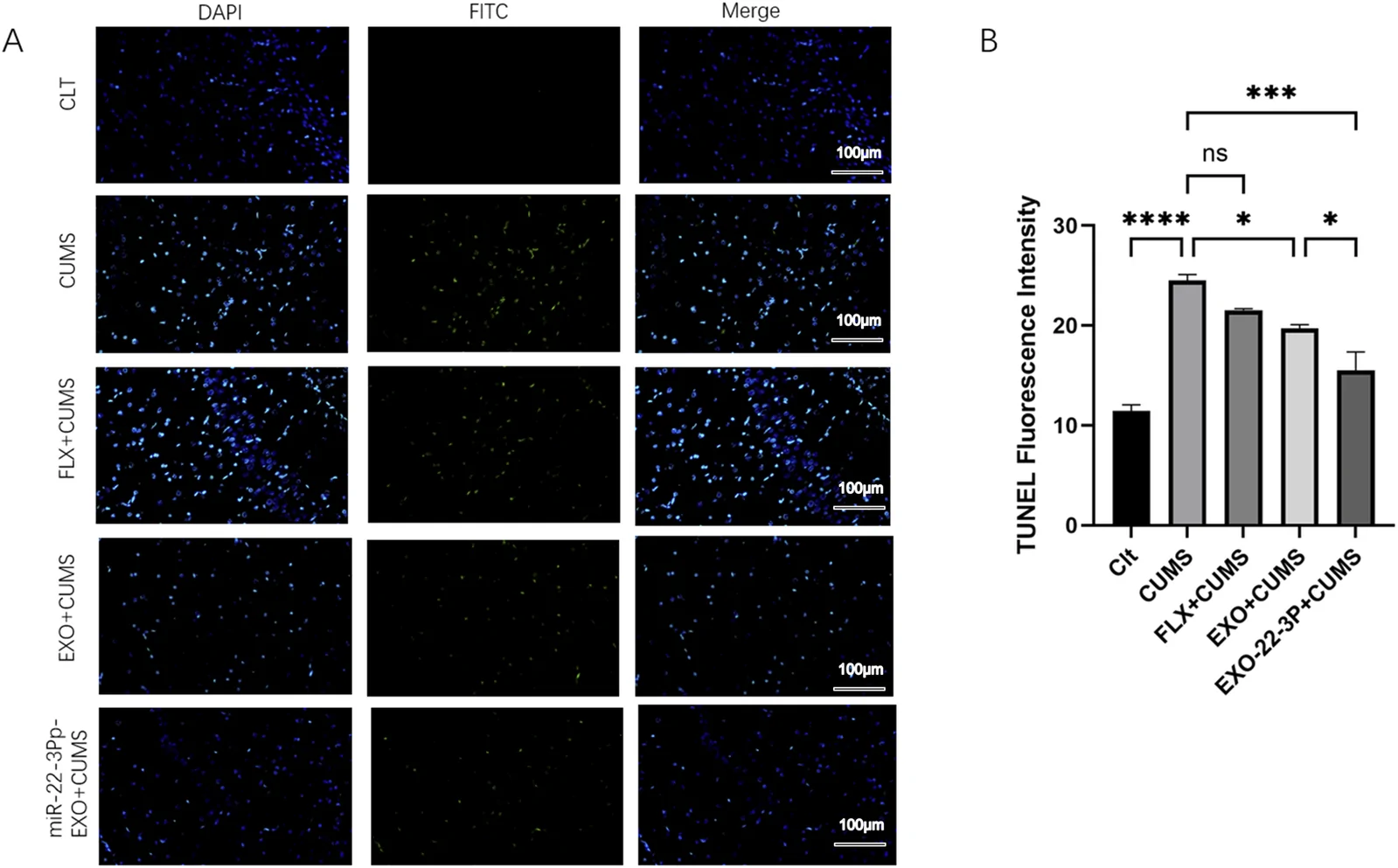
Effects of Exosomes and Fluoxetine on Neuronal Apoptosis in the Rat Hippocampus. **(A)** TUNEL staining of hippocampal neurons in rats from each group (×400). The DAPI channel shows blue nuclear staining; the FITC channel shows apoptotic nuclei; Merge indicates the merged image. **(B)** Comparison of neuronal apoptosis intensity in the hippocampal tissue of rats among groups.

These results suggest that engineered exosomes exert a stronger anti-apoptotic and neuroprotective effect in the hippocampus of CUMS-induced depressive rats.

## 4 Discussion

Mesenchymal stem cell-derived exosomes have emerged as promising therapeutic carriers due to their regenerative and immunomodulatory properties (Auger and Kajimura, 2023; Sumarwoto et al., 2021). Studies have shown that stress can trigger activation of the hypothalamic–pituitary–adrenal axis, increase corticosteroid levels, and downregulate hippocampal neurogenesis. Depression is associated with impaired hippocampal plasticity, including increased neuronal apoptosis and reduced neurogenesis (Ren and Guo, 2021). Proliferation, differentiation, and apoptosis are continuous processes in adult hippocampal neural cells. Numerous studies have demonstrated that depression and stress can induce hippocampal cell apoptosis in rodents, non-human mammals, and humans (Lucassen et al., 2006). Evidence indicates that repeated separation of pups from their mothers leads to increased apoptosis in the dentate gyrus of the hippocampus, along with impaired memory and depression-like behavioral changes (Sung et al., 2010). Depression is associated with impaired hippocampal plasticity, including increased neuronal apoptosis and reduced neurogenesis.

MicroRNAs are important regulators of gene expression and have been implicated in neuroinflammatory and neurodegenerative processes. Among them, miR-22-3p has been reported to exert anti-inflammatory and neuroprotective effects (Mishra, 2014; Warnecke-Eberz et al., 2015). Exosomes are natural nanocarriers capable of crossing the blood–brain barrier and delivering bioactive molecules, making them promising candidates for central nervous system-targeted therapies (Andre et al., 2024). Intranasal administration represents a non-invasive route that enables direct transport of exosomes to the brain, bypassing the blood–brain barrier. Compared with systemic delivery, this approach improves brain targeting efficiency (Riazifar et al., 2019; Wen et al., 2019; Xie et al., 2019).

Mechanistically, the interaction between engineered exosomes and neuronal cells in depression is complex. In this study, we identified the FURIN/HMGB1 axis as a potential pathway involved in miR-22-3p-mediated effects. Suppression of FURIN was associated with reduced HMGB1 expression, decreased neuroinflammation, and attenuation of neuronal apoptosis. In the present study, intranasally administered ADMSC-derived exosomes efficiently crossed the blood–brain barrier and improved depressive-like behaviors in rats. Notably, miR-22-3p-engineered exosomes exhibited enhanced therapeutic efficacy compared with native exosomes. Moreover, engineered miR-22-3p-EXO exerted a more pronounced ameliorative effect on depression-like behaviors. Histological analyses (HE and TUNEL staining) revealed that engineered exosome treatment reduced neuronal apoptosis and preserved neuronal morphology in the hippocampus and prefrontal cortex. These findings suggest that miR-22-3p-engineered exosomes exert antidepressant effects by inhibiting neuronal apoptosis and attenuating neuroinflammation.

Mechanistically, the interaction between engineered exosomes and neuronal cells in depression is complex. In this study, we identified the FURIN/HMGB1 axis as a potential pathway involved in miR-22-3p-mediated effects. Suppression of FURIN was associated with reduced HMGB1 expression, decreased neuroinflammation, and attenuation of neuronal apoptosis. In summary, this study demonstrates that intranasal delivery of miR-22-3p-engineered exosomes effectively alleviates depressive-like behaviors, likely through modulation of the FURIN/HMGB1-mediated neuroinflammatory and apoptotic pathways. These findings highlight a novel and non-invasive therapeutic strategy for depression. Intranasal delivery represents a non-invasive and efficient strategy for targeting the central nervous system, bypassing the blood–brain barrier and enhancing therapeutic efficacy. This delivery route bypasses the blood–brain barrier, enhances central nervous system targeting efficiency, and minimizes systemic exposure, thereby improving therapeutic outcomes and reducing potential side effects. Compared with direct stem cell transplantation, exosome-based therapy offers several advantages, including lower immunogenicity, reduced risk of tumorigenicity, improved biosafety, and easier storage and delivery. These properties make exosomes particularly attractive as a cell-free therapeutic strategy in regenerative medicine. These findings highlight the therapeutic potential of MSC-derived exosomes as a novel cell-free strategy for treating depression.

## Data Availability

The original contributions presented in this study are included in the article and its Supplementary Material. Further inquiries can be directed to the corresponding authors.
